# Estimating the Bariatric Surgery Rate in the Potentially Eligible Population within the Brazilian Public Healthcare System, 2017–2023

**DOI:** 10.1007/s11695-026-08720-3

**Published:** 2026-05-13

**Authors:** Luis Roberto da Silva, Claudio Makoto Kanai, Anna Carolina Batista Dantas, Wayner Vieira de Souza, Denis Pajecki, Beatriz Helena Tess

**Affiliations:** 1 Departamento de Medicina Preventiva, Faculdade de Medicina da Universidade de São Paulo, São Paulo, Brazil; 2 Departamento de Gastroenterologia, Faculdade de Medicina da Universidade de São Paulo, São Paulo, Brazil; 3 In memoriam. Departamento de Saúde Pública, Instituto Aggeu Magalhães - Fundação Oswaldo Cruz, Recife, Pernambuco, Brazil; 4 Unidade de Cirurgia Bariátrica e Metabólica, Hospital das Clínicas HCFMUSP da Faculdade de Medicina da Universidade de São Paulo, São Paulo, Brazil

**Keywords:** Obesity, Bariatric Surgery, Health Services Accessibility, Public Health, Brazil.

## Abstract

**Introduction/Purpose:**

Access to bariatric surgery (BS) remains limited in most countries with public healthcare systems, despite the continued rise in obesity prevalence, and Brazil is no exception. This study aimed to estimate the BS rate among adults with severe obesity who were potentially eligible for the procedure in Brazilian state capitals and the Federal District between 2017 and 2023.

**Methods:**

Publicly available national databases were used to obtain the number of performed surgeries and to estimate the eligible population. Severe forms of obesity were defined as body mass index (BMI) ≥ 40 kg/m² or BMI between 35 and 40 kg/m² in the presence of diabetes or hypertension. The BS rate was calculated using, as the denominator, the number of adults with severe forms of obesity who reported having no private healthcare insurance.

**Results:**

1,251,075 adults living in the study sites had severe forms of obesity. Of these, 738,430 reported having no private healthcare insurance. Between 2017 and 2023, 8,205 BS were performed on residents who underwent the procedure in their state capital. Overall, only 1% (95% CI 1.09–1.14) of eligible individuals underwent BS in the Brazilian Unified Health System (SUS) over the study period.

**Conclusion:**

These findings highlight the extremely limited access to surgical treatment for severe forms of obesity in the SUS, despite the country being a global leader in the absolute numbers of BS. Accurate estimates of the number of potential candidates for BS may help identify barriers to treatment which may, in turn, increase the BS rates in Brazil and elsewhere.

## Key Points

We estimated bariatric surgery rates among potentially eligible adults.

We used public databases to estimate both the numerator and denominator.

The bariatric surgery rate in the Brazilian state capitals and the Federal District was 1%.

Accurate estimates of eligible adults may help identify barriers to surgical treatment of obesity and increase the BS rates.

## Introduction

According to the International Federation for the Surgery of Obesity and Metabolic Disorders (IFSO) [1], Brazil performs the second-highest absolute number of bariatric surgeries (BS) worldwide, after the United States of America. However, although a total of 80,441 BS were recorded in Brazil in 2023, only 7,570 (9.4%) of these were performed in the public Brazilian Unified Health System (SUS); the remaining procedures were performed through private health insurance or self-payment [2]. This disparity underscores the limited access to BS in the public sector, despite the fact that most Brazilians rely exclusively on this system for healthcare [3, 4]. Hospitals which provide BS within the SUS have long waiting lists of patients who may have to wait for years for treatment.

To quantify the gap between the demand for, and the availability of BS, in the SUS, it is essential to estimate the number of individuals who meet the eligibility criteria for the procedure. This study aimed to estimate the rate of BS among the potentially eligible population residing in Brazilian state capitals and the Federal District between 2017 and 2023.

## Methods

This study used publicly available data. The number of surgeries performed between 2017 and 2023 was obtained from the Hospital Information System of the SUS Informatics Department (DATASUS) [5]. Inclusion criteria were adults aged 19–65 years residing in the 26 Brazilian state capitals or the Federal District. Data were extracted using the microdatasus package [6] in RStudio (version 4.2.3).

Severe forms of obesity were defined as body mass index (BMI) ≥ 40 kg/m² or BMI between 35 kg/m² and 40 kg/m² associated with diabetes or hypertension. This definition was used to describe the eligible population. We estimated the number of individuals eligible for surgery based on the prevalence of severe forms of obesity derived from the Surveillance System for Risk and Protective Factors for Chronic Diseases by Telephone Survey (Vigitel) [7]. Prevalence estimates were restricted to adults aged 19–65 years to ensure comparability between the numerator and the denominator. We calculated the BS rate by dividing the number of surgeries by the number of individuals with severe forms of obesity who reported no private health insurance, expressed per 100 individuals.

The Hospital Information System database is based on the Hospital Admission Authorization, an administrative document required for reimbursement of hospitalizations funded by the SUS. This database contains information on the primary procedure performed, patient municipality of residence, hospital location, and other demographic and administrative variables. Vigitel collects self-reported information on health conditions and risk factors, including private health insurance usage and self-reported weight and height, which are used to calculate BMI.

The survey employs probabilistic sampling of adults residing in the Brazilian state capitals and the Federal District. Because landline telephone coverage varies across cities, Vigitel applies post-stratification weighting using the Rake method to adjust the sample distribution to match sociodemographic characteristics of the adult population (sex, age group, and education level), according to official population estimates. These weighting procedures aim to enhance representativeness despite the inherent limitations of telephone-based surveys [7, 8].

Hospital admissions for BS were identified in the Hospital Information System using records with obesity (ICD-10 code E66) as the main diagnosis and the following procedures in the “performed procedures” field: gastrectomy with or without duodenal switch, gastroplasty with intestinal bypass, vertical gastroplasty with band, sleeve gastrectomy, and laparoscopic BS.

To ensure consistency with the population-based estimates derived from Vigitel, we included only hospitalizations of individuals whose municipality of residence corresponded to the capital city where the surgery was performed. Thus, patients who travelled from other municipalities to undergo surgery in the capital were excluded from the analyses.

The R codes and other materials used are available on the Open Science Framework Repository: https://osf.io/wbdav/overview?view_only=c1c36cbdc2224b17a28f01ed8605f606.

Ethical approval was not required because the study used only publicly available anonymized data. ChatGPT and Gemini were used solely to assist with R code development and language editing. All outputs were critically reviewed and validated by the authors to ensure the integrity and originality of the manuscript.

## Results

**Fig. 1:**
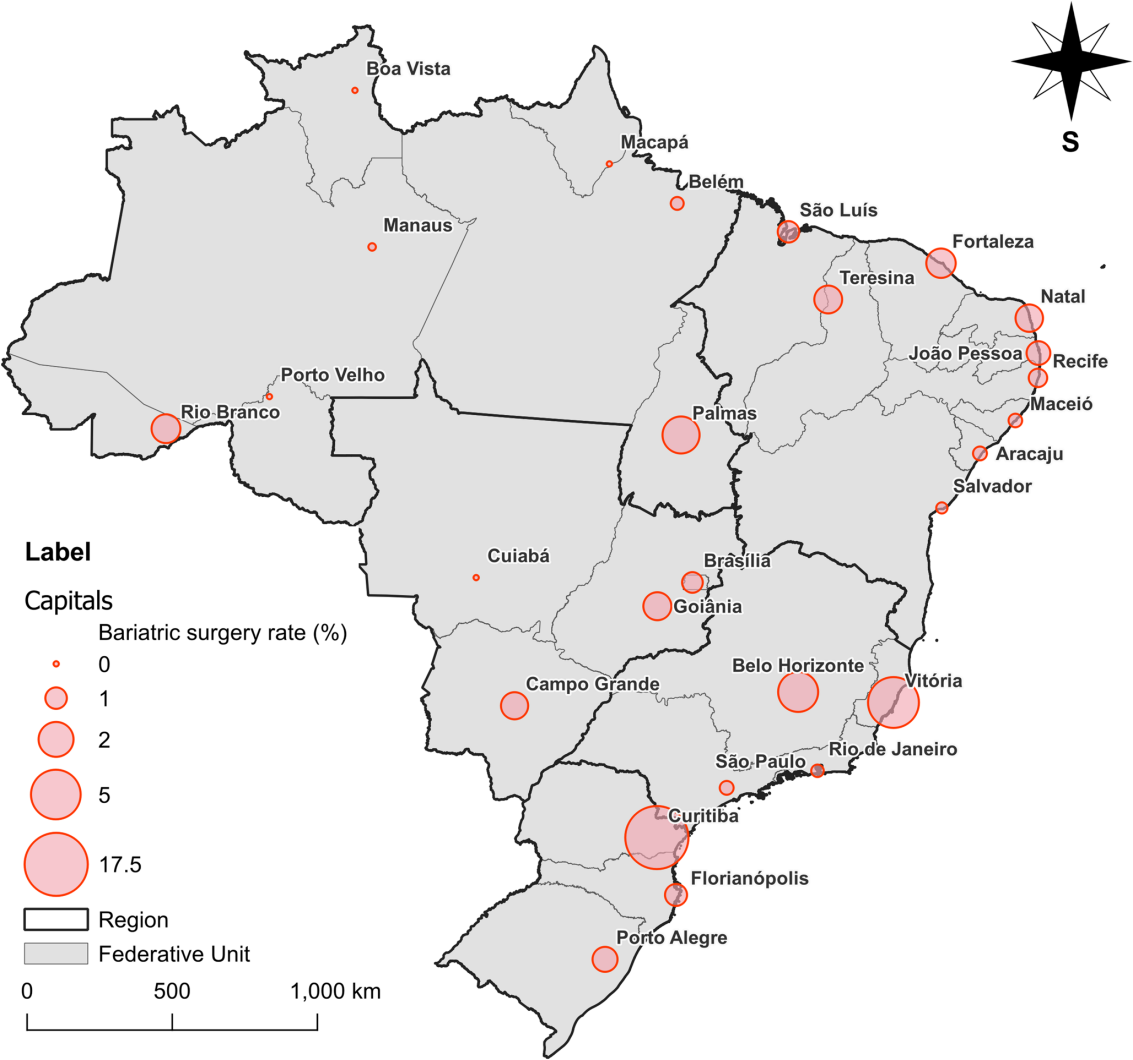
Bariatric surgery rates among adults with severe forms of obesity and no private healthcare insurance in the Brazilian state capitals and the Federal District, 2017–2023

The estimated total number of adults with severe forms of obesity living in the Brazilian state capitals and the Federal District was 1,251,075. Of these, 738,430 individuals reported having no private health insurance. This population was considered the denominator for the rate calculation. Between 2017 and 2023, a total of 8,813 BS were performed in the SUS among residents of Brazilian state capitals aged 19–65 years. Of these, 608 individuals underwent surgery in other municipalities and were excluded from the analyses. Only those who underwent surgery in the capital of their home state were included as the numerator, totaling 8,205 procedures. The overall BS rate among eligible individuals in the SUS was 1% (95% CI 1.09–1.14), representing cumulative access over the study period. Capitals located in the South and Southeast regions showed higher surgical rates, while those in the North exhibited the lowest rates. Curitiba (capital of the state of Paraná) had the highest surgical rate (17.51%). Four capitals had no BS recorded among their residents during the study period: Boa Vista (Roraima), Cuiabá (Mato Grosso), Macapá (Amapá), and Porto Velho (Rondônia) (Fig. 1; Table 1).

**Table 1:** Bariatric surgery rates (and 95% confidence intervals) among adults with severe forms of obesity and no private healthcare insurance in the 26 Brazilian state capitals and the Federal District, 2017–2023

Capital	State	Number of surgeries^*^	Estimated eligible individuals^**^	BS rate per 100 individuals (95% CI)
Central-West region		550	51,705	1.06 (0.98, 1.16)
Brasília	Distrito Federal	131	13,968	0.94 (0.79, 1.11)
Campo Grande	Mato Grosso do Sul	122	8,723	1.40 (1.17, 1.67)
Cuiabá	Mato Grosso	0	8,705	0
Goiânia	Goiás	297	20,309	1.46 (1.31, 1.64)
Northeast region		1,490	175,658	0.85 (0.81, 0.89)
Aracaju	Sergipe	44	10,692	0.41 (0.31, 0.55)
Fortaleza	Ceará	356	22,857	1.56 (1.40, 1.73)
João Pessoa	Paraíba	148	12,753	1.16 (0.99, 1.36)
Maceió	Alagoas	85	21,657	0.39 (0.32, 0.49)
Natal	Rio Grande do Norte	194	13,666	1.42 (1.23, 1.63)
Recife	Pernambuco	302	39,093	0.77 (0.69, 0.86)
Salvador	Bahia	62	28,536	0.22 (0.17, 0.28)
São Luís	Maranhão	184	18,469	1.00 (0.86, 1.15)
Teresina	Piauí	115	7,935	1.45 (1.21, 1.74)
North region		244	79,657	0.31 (0.27, 0.35)
Belém	Pará	69	20,515	0.34 (0.27, 0.43)
Boa Vista	Roraima	0	9,662	0
Macapá	Amapá	0	7,858	0
Manaus	Amazonas	9	24,328	0.04 (0.02, 0.07)
Palmas	Tocantins	57	2,575	2.21 (1.71, 2.86)
Porto Velho	Rondônia	0	7,558	0
Rio Branco	Acre	109	7,161	1.52 (1.26, 1.83)
Southeast region		2,384	376,160	0.63 (0.61, 0.66)
Belo Horizonte	Minas Gerais	877	34,140	2.57 (2.41, 2.74)
Rio de Janeiro	Rio de Janeiro	274	88,001	0.31 (0.28, 0.35)
São Paulo	São Paulo	1,017	250,014	0.41 (0.38, 0.43)
Vitória	Espírito Santo	216	4,005	5.39 (4.74, 6.14)
South region		3,537	55,250	6.40 (6.20, 6.61)
Curitiba	Paraná	3,084	17,616	17.51 (16.95, 18.08)
Florianópolis	Santa Catarina	84	8,080	1.04 (0.84, 1.29)
Porto Alegre	Rio Grande do Sul	369	29,554	1.25 (1.13, 1.38)
Total		8,205	738,430	1.11 (1.09, 1.14)

^*^Number of surgeries performed in the Brazilian Unified Health System (SUS)

^**^Estimated number of individuals with severe forms of obesity and no private healthcare insurance. Severe forms of obesity were defined as BMI ≥ 40 kg/m² or BMI between 35 kg/m² and 40 kg/m² associated with diabetes or hypertension

## Discussion

To our knowledge, this is the first study to use multiple national public databases to estimate BS rates among individuals with severe forms of obesity who are potentially eligible for surgery in the public healthcare system in Brazil.

In 2025, according to data from the Brazilian National Supplementary Health Agency, approximately one quarter of the Brazilian population was covered by private medical health insurance, corresponding to about 53.3 million individuals. This figure indicates that around three quarters of Brazilians depend exclusively on the SUS [9].

We observed that, overall, only 1% of adults potentially eligible for BS underwent the procedure in the SUS over the study period (2017–2023). Although Brazil is a global leader in absolute surgical volume, coverage among eligible adults in the SUS remains extremely low. Two studies conducted in the periods 2008–2018 [3] and 2009–2019 [4] reported BS rates in the SUS of 4.86 and 2.44 per 100,000 inhabitants in Brazil, respectively. However, unlike the present study, these analyses used the general population as the denominator, without considering BMI or surgical eligibility criteria. This methodological difference may have led to an underestimation of the BS rates, since this procedure is indicated for specific populations with obesity and associated comorbidities.

Limited access to BS is not unique to Brazil. Other countries, particularly those with universal healthcare systems, face similar challenges. In New South Wales, Australia, between 2013/14 and 2021/22, a rate of 150.1 BS per 100,000 adults was reported; however, this estimate used the general population as the denominator and did not account for obesity status [10]. In Canada, between 2012 and 2013, the rate of BS was 5.4 per 1,000 individuals with a BMI ≥ 35 kg/m², indicating that only 0,54% of eligible individuals underwent the procedure [11]. In the United States of America, BS among adults with severe obesity was approximately 1% in 2015 [12]. These findings highlight that, regardless of the healthcare system model, the rate of BS remains consistently low when compared to the potential demand among individuals with severe obesity.

Marked regional disparities were observed in BS rates in Brazil. Although all state capitals and the Federal District had potential demand for BS, four capitals, three of which are in the North region, recorded no surgeries during the study period. In contrast, Curitiba, in the South region, recorded the highest surgical rate, 17.51%. Previous studies have documented this unequal distribution, with higher BS rates concentrated in states in the Southeast and South regions and lower rates in the North of the country [3, 4]. These differences are associated with the structural capacity of the health system which varies across the regions. Limitations within the health system include the availability of specialized human resources, adequate hospital infrastructure, equipment, material resources, and financial support [3]. These issues are not only associated with the regional disparities, but also contribute to the low BS rates observed in this study [11, 13, 14].

Accurate estimates of the current level of BS in Brazil will help to identify the most urgent actions needed to improve access in the SUS. These actions should be coordinated across multiple sectors. Updating national clinical guidelines to reflect expanded eligibility criteria based on current evidence is essential [15]. In addition, it is necessary to expand, in a decentralized and regionalized manner, the number of hospitals authorized to perform BS in the SUS. It is also critical to strengthen continuing health education among primary health professionals to indicate surgery and to identify potential candidates while bearing in mind safety issues of the procedures. Furthermore, improving the care pathway in the SUS, enhancing digital health strategies for patient follow-up when feasible, and incorporating interprofessional approaches to obesity care into undergraduate health education are key strategies to increase the access to BS in the SUS.

A further consideration for future obesity care relates to the use of new pharmacological treatments, such as the glucagon-like-peptide 1 receptor agonists (GLP1 RA). Despite a rapid increase in the use of these drugs, BS remains, so far, the most cost-effective option and it provides sustained long-term weight loss for the treatment of severe forms of obesity [10, 16, 17]. Although pharmacological treatments are highly effective, their long-term influence on the demand for BS is unclear. In Brazil, such treatments are not available in the SUS, and this reinforces the importance of expanding access to surgical treatment.

This study has several limitations. First, the research question was formulated a posteriori and was not directly aligned with the original objectives of the data collection. Nevertheless, the integration of three public databases made it possible to address the proposed question, representing a methodological advance in the investigation of access to BS in the SUS. Second, estimates of the population with obesity derived from Vigitel may be subject to information bias, as individuals with obesity tend to underestimate their body weight [18]. Telephone surveys may also underrepresent individuals from lower socioeconomic strata who have limited access to landline telephones. However, Vigitel employs probabilistic sampling and post-stratification weighting procedures to help ensure the representativeness of the adult population residing in Brazilian state capitals and the Federal District [8]. Despite these limitations, Vigitel remains an important surveillance system for chronic disease risk factors in Brazil. Third, for the definition of eligibility, only hypertension and diabetes were considered as comorbidities associated with grade II obesity. Other relevant clinical conditions were not available in the database, which may have led to an underestimation of the number of eligible individuals and, consequently, of surgery rates. Fourth, we used the proportion of individuals covered by private healthcare insurance as a proxy to estimate the population dependent exclusively on the SUS in the state capitals and the Federal District. Although this strategy does not allow for the precise identification of the proportion of individuals with severe forms of obesity who would effectively use the public healthcare system, it represents the best methodological approximation currently possible based on the available data sources.

## Conclusion

We observed a very low BS rate of 1% in the public healthcare system in the 27 Brazilian state capitals and the Federal District. Our study advances the calculation of BS rates by using public population-based datasets to provide a more accurate estimate of the number of potentially eligible individuals in the SUS. However, further advances in the methodological approaches and population-based surveys will be necessary to capture data on patients refusing BS or having the indication of other treatments. Expanding policies aimed at decentralizing and regionalizing access to BS services is essential to reduce geographic disparities. Addressing barriers to surgical treatment of obesity may increase the BS rates in Brazil and elsewhere.

## Data Availability

All data used in this study are publicly available in the original databases cited and have been consolidated in the study repository. The R codes and other materials used are available on the Open Science Framework repository: https://osf.io/wbdav/overview?view_only=c1c36cbdc2224b17a28f01ed8605f606.
